# The Packing of Amalgam, with a Review of Some Literature upon the Subject1Read before the Harvard Odontological Society, Boston, May, 1905.

**Published:** 1905-08

**Authors:** Herbert Locke Wheeler

**Affiliations:** New York City


### THE PACKING OF AMALGAM, WITH A REVIEW OF SOME LITERATURE UPON THE SUBJECT.[1]

BY HERBERT LOCKE WHEELER, D.D.S., NEW YORK CITY.

It is with hesitation that I assume to speak upon the subject of amalgam, because the amount of scientific knowledge we possess upon the subject is so meagre that, though there are pages and

---

[1] Read before the Harvard Odontological Society, Boston, May, 1905.

pages of matter to be found touching upon the subject, the valuable information contained therein seems to be very slight, and none of them seem to possess the virtue of having obtained scientific data from a series of reported observations upon the behavior of this material in the mouth for a long period of years.

Considering the effectual quiescence apparently placed upon further effort in this line of investigation by the series of papers upon this subject written by Dr. Black, which appeared in the *Dental Cosmos* in 1895–96, it is with some trepidation that I now address this dignified body of *confrères.* It seems to me that there is a feeling abroad among many that these papers contained about all that is knowable upon the subject,—past, present, and future. For my own part, I am amazed that such a cataract of words which contain such a mere rivulet of thought should receive the plaudits of men who seem to have some considerable reputation for discrimination and sense. Anyway, there seems to have been a marked temerity in addressing ourselves to this problem.

In order to get at a suitable understanding of what I shall say concerning the knowledge of the behavior of amalgams in the mouth and the processes of insertion, I shall quote freely from various authors who have written upon this subject both here and abroad.

You are doubtless all so well acquainted with the experiments of Drs. Bogue and Hitchcock, who were connected with Harvard, that I shall not dwell much upon their experiments. Of all the early experiments in this field, it seems to me that Dr. J. Foster Flagg accomplished the most in introducing this material and producing known and useful formulæ; also in discovering the various qualities of amalgams.

This seems to have been to some extent due to his greater independence of action and freedom from association with dental supply houses. I fancy Dr. Hitchcock made to some extent the mistake that is apparent in some of Dr. Black's work of giving too much credence to the statements of interested parties.

The principal properties that it has been desirable to overcome are shrinkage and the tendency to change form,—called spheroiding by some, and "flow" by others,—and much work has been expended in trying to find the peculiarities of various combinations of metals that would produce good amalgams and perfect fillings. In this Flagg's work stood out pre-eminently, and he probably produced as good alloy for filling-material as any of the more modern and more heralded alloys of secret formulæ that have been made possible by these wonderful scientific(?) papers of Dr. Black.

The unfortunate comparison made at the beginning of this series of papers would seem to indicate confusion in the mind of the author as to the difference between operating upon a physical, sensitive, living organism, with its temperamental peculiarities, and upon inanimate wood, stone, and iron, and he does not seem to have observed that a comparison between intermittent force of muscular derivation and steady strain of known weight from the force of gravity hardly constitutes a reasonable analogy. However, this paragraph is quite in keeping with the wilderness of words that one is forced to wander through in order to get what little there is of value in the articles. The remarks upon stress and the necessity for great strength in a filling-material in order to avoid bulging could be more easily accepted if the fillings of various qualities of gutta-percha were not often known to stand up well, even when it has to withstand the force of mastication. If a tooth does not bear upon it harder than upon the other surfaces during repose, much wear will often take place from the grinding process without causing the least particle of bulging. This seems to me pretty good evidence that Professor Black's theory of stress in untenable. Furthermore, how common it is to find amalgam fillings in buccal cavities of molars quite as badly creviced and changed in form as compound fillings upon the approximal and grinding surfaces. Dr. Black says that the past literature upon the subject has in no wise been ignored by him, but this is a vague statement, and a careful reading of the papers would indicate that further reading could have been done by him with considerable advantage.

Mr. Charles Tomes, of England, who is probably not surpassed in the breadth of his learning and the accuracy of his observations by any one in our calling, has remarked that Dr. Black did not quote anybody's researches, as a general rule, and that a fault of his paper was that he stated the whole question *tabula rasa.*

It is not impossible that Dr. Black's notes of thanks for favors received occupied much of his time, for he says, "I wish to thank manufacturers of dental goods for the hearty support they have given me in this work. When it was announced to them that I would undertake an investigation of the physical properties of amalgam, I received within a few weeks about eighty different alloys (apparently it was known to the manufacturers what the investigation was to be long before it was known to the profession). A number of manufacturers have also given me their formulæ, thus enabling me to conduct the investigation on *purely scientific lines.* These formulæ are trade secrets which must be respected." This, then, is the wonderful scientific spirit of these articles, started in the very beginning by giving a first mortgage to the commercial interests. Verily this is giving unselfish service to the dental supply houses.

Professor Flagg described the change that takes place in an amalgam filling after its insertion as bulging, which he attributed to spheroiding, and, according to Dr. St. George Elliott, Dr. J. Smith Dodge first promulgated this theory many years ago,—that is, a tendency of the mercury in the mass to form globules; but this explanation has by no means been proved, and I think is not generally accepted by those who have been investigating. It is, however, worthy of notice that Dr. Flagg stated that this change is more pronounced in alloys composed of metals of more yielding texture, less obdurate in melting, and more deliberate in cooling, and this quality in amalgams is what produces crevicing, sometimes called the "black ditch."

Professor Black describes the same quality, and attributed it to stress. He called this quality "flow," and maintained that an increase in stress is required to continue the "flow" after the area of amalgam had been greatly increased by the flattening of the mass, but that it has to receive pressure slowly, as a silver-tin amalgam is not malleable. It is well to notice that in one of his experiments, in which the formula given is silver, 47.06; tin, 51.76; copper, 0.94; and zinc, 0.24, it was mixed with mercury in a mortar. The mass was divided into various parts, some in which the excess of mercury was removed, and some in which it was not. Of these, the block that showed the least flow was one in which the amalgam had been wrung through muslin to squeeze out the excess of mercury. The filling from this mass was made with mallet force, all the mercury possible being removed in the filling process. The filling which was made in this way showed a flow of about seventeen per cent. in one hour, while some of those in which the excess of mercury was not removed the flow was as high as eighty-nine per cent.

In my estimation, this is far from substantiating Dr. Black's claim that the best way to pack amalgam is with a serrated instrument, avoiding the removal of mercury. On the contrary, it seems to be evident that the chief necessity in obtaining good amalgam fillings is to remove all the mercury possible.

Dr. Black also claims that the strength of the mass depends mostly on the perfect evenness of the distribution of the mercury. He does not seem to have considered that the size and shapes of the crystals which form in the setting process may have an important bearing upon the strength of the filling-materials, and claims that after mercury is mixed with alloy, and evenly distributed, any form of violence weakens the product, as, for instance, the burnishing process after crystallization has commenced. I do not see that he has produced one iota of evidence to prove this.

Mr. Amos Kirby, of England, of whom Dr. Black seems never to have heard, contends that there is a rearrangement of the mercury through the mass of amalgam after the filling is completed, to which rearrangement Mr. Kirby ascribes the warpage, as he chooses to call it, of amalgam fillings, and he has apparently demonstrated with blocks of amalgam that he can prevent their contraction and keep them straight or can make them bend one way or the other, according to the distribution of mercury in the mass. Mr. Tomes has found by the use of the microscope that the setting of an amalgam is a process of crystallization, and that the question of the quantity of the mercury in mixing the amalgam made no material difference in the crystals or rhombs, and he decided that the reason for an amalgam filling having a mat surface, as it were, when fully hardened, although it had been carefully burnished in the filling process, is due to this crystallization. He also discovered that the crystallization takes place so that the crystals or rhombs stand up from all surfaces of the filling in such a way as to make the surface look like pavement epithelium, or, on a minute scale, furry.

He found the size of the crystal to differ greatly in different amalgams. In Welch's amalgam,—tin, 51.52; silver, 48.48,—which Mr. Tomes used in his experiment, the rhombs were one-twelve-hundredth to one-eighteen-hundredth of an inch in diameter and about twice as long as they were wide.

In standard amalgam, made by Eckfelt and Du Bois,—silver,

52; tin, 40.60; gold, 4.40; copper, 3,—they are about half as large, and with an amalgam made of precipitate of silver they are smaller yet. Mr. Tomes thinks that it may be the sprouting of crystals over the surface of amalgam that failed to show expansion by specific gravity tests that may cause the breaking of glass tubes in which they are packed, but he suggests that it is not certain that the crystals do sprout up—it may be that the intermediate portions sink. Mr. Tomes also finds that these crystals are plastic, and suggests that the quality called "flow" by Dr. Black, which he determined by pressing a foreign substance against an amalgam by the pressure of a strong spring was accounted for in this way.

Mr. Tomes further adds, of the so-called flow: "I do not believe that it has, as supposed by Dr. Black, anything to do with the ultimate failure of amalgams, for I doubt if the very intermittent pressure to which they are subjected would bring it about."

Dr. Black speaks of alloys that were cut very fine, as showing a great amount of flow, and speaks of coarse-cut alloy taking up less mercury in mixing than fine cut.

Dr. J. Morgan Howe called attention to this some years before, —January, 1894, *Transactions of the New York Odontological Society*,—but no one seems to have noticed it.

Dr. Black states that the loss in weight in wringing an amalgam in cloth was purely the mercury. This is not indicated by the experiment of Mr. Tomes, who says, speaking of expressed mercury that he pressed from an amalgam mix which he had put under a glass slide for examination by the microscope, "No rhombs appeared, but only foliaceous forms similar to those in which tin crystallizes." From which it appears that tin is carried off by the mercury in the squeezing-out process. Dr. Black shows, in his first paper, experiments with alloys with sixteen different formulæ, and he states immediately under this table that the formula of the alloy controls in a large degree the flow of the amalgam formed. He states, also, that every silver-tin amalgam in the market is, when mixed with a low percentage of mercury, so strong that they will not break down or crush, and yet he assumes that what he is pleased to call the "black ditch" about the margins is caused by the stress of mastication. I have never yet seen an amalgam filling made after the so-called "Black method" with modern alloys that did not show crevicing at the edge. And while stating, as Dr. Black does, that the product of the manufacturers is quite all that is necessary for the profession, he says in his last paper that what the dental profession and the men who supply its members need to know is the conditions of contraction and expansion of amalgam, including the formula of alloy and whatever else may influence. In comparison with this statement I wish to read a letter from Dr. Black to the S. S. White Dental Company, which has been published, and which is hardly in keeping with the statements in these papers.

"CHICAGO, April 16, 1905.

"MR. C. L. BINGHAM, Chicago:

"DEAR SIR,—Your note of the 7th inst. has remained unanswered for reason that I was out of town. You inquire as to the matter of the advertising of alloys purporting to be made in strict accord with Dr. G. V. Black's formula. I have become aware that some such advertisements were being made use of wrongfully. No one has any such right from me. Furthermore, I have no formula by which a thoroughly reliable alloy can be made by any one. Any one who knows the methods which I have developed, and has made a sufficient study of the metals to enable him to make alloys understandingly, needs no formulæ. Even then he will be unable to make reliable alloys without the special instruments developed for that purpose; for he will know that uniform alloys cannot be made from any fixed formula. Therefore, any advertisement setting forth that any given alloy is made according to my formula is without authority from me, and is a fraud.

"Certain persons, all of whom you know, are making alloys after the methods which I have developed, they having prepared themselves for so doing by a regular course of study and technical drill and providing themselves with the necessary apparatus.

"Very truly,

"G. V. BLACK."

Dr. Black's contention, that in amalgams which show both contraction and expansion the contraction is in the upper half of the filling and expansion in the lower, is quite in keeping with some of Mr. Kirby's experiments, and also an evidence that the method of packing amalgam filling is important, and the most important part of the process is to remove the mercury to such an extent that the upper surface is as dry, when completed, as the lower.

Dr. Black's position in protecting these wonderful secret formulæ and ignoring the plain ethical demands of his profession, by upholding secret and proprietary formulæ, is only a part of that influence that is commercializing the world to-day to the detriment of the individual, and in so far as he has done this it seems to me he has contributed not to the advancement of our profession, but to its degradation.

And after all of this laborious work done for our kind benefactors, the manufacturers, behold the wonderful result! He concludes that a formula composed of silver, 68.5; tin, 25.5; gold, 5; zinc, 1, has been watched long enough to assure its success.

This statement appeared in December, 1896. When I was in college in 1889–90 the formula for an especially hard contour amalgam which Professor J. Foster Flagg was teaching to his students was silver, 70; tin, 26; gold, 3; zinc, 1. Not much difference in these, is there? And the original formula, of which this was a modification,—silver, 68; tin, 27; gold, 4; zinc, 1,—was brought out by Dr. Flagg in 1884, twelve years before this wonderful discovery.

Now let us consider the approximate formulæ of some of the modern alloys, bearing in mind that they are all heralded as Black's method or formula. These alloys were analyzed by Booth, Garrett, and Blair, of Philadelphia; Fellowship alloy, silver, 67.73; tin, 26.33; copper, 4.71; zinc, 1.23. Rego alloy, silver, 66.54; tin, 28.14; copper, 4.21; zinc, 1.06. Triumph alloy, silver, 66.74; tin, 27.44; copper, 4.24; zinc, 1.42. Twentieth Century, silver, 66.81; tin, 27.32; copper, 4.39; zinc, 1.51. Wonderful, is it not? All of these marvellous, perfect modern, scientific, *à la* G. V. Black dental alloys have succeeded in eliminating the one expensive metal, gold, from their formulæ, and yet raise the price higher than formerly, and are highly commended by their worthy benefactor as furnishing alloys of the "purest of metals."

And this is the practical outcome, as far as dentists are concerned, of the experiments of Professor Black. It is well to place here the words of our British cousin, Mr. Tomes, when we are prone to pat ourselves upon our backs because of our superior knowledge of dentistry. He says, "I believe that an enormous amount of experience in the hands of countless operators has been entirely wasted through our being content to use amalgams, the composition of which is unknown to us. For my own part, since I have been experimenting I have resolved to use no amalgam the composition of which is not known to me, and I think the manufacturers would do well to always publish it on the bottles."

Dr. Black's statement, that alloys cut very fine make a soft, pasty mass, and work smoothly and easily, while those that are cut coarse make a mass that is not so pasty, and that does not work so smoothly, is not at all in accordance with the careful observation of Dr. J. Morgan Howe, given to the New York Odontological Society in the paper before mentioned.

Dr. Howe found, in the coarse filling and the fine filling of the same batch of any given alloy, using the same quantity of mercury for mixing each, that the coarse fillings made by far the most plastic mass, while these same fillings mixed with thirty-three and one-third per cent. less mercury than the fine gave about the same plasticity,—so nearly the same that the difference was not discernible,—and that after several hours the filling made from the coarse-grain alloy seemed to be much harder. Dr. Howe, after years of observation of the behavior of fillings in the mouth, has come to think that the addition of zinc in any quantity to an alloy for amalgams is probably a disadvantage, and deleterious to the filling. My own experience in the working of amalgams is that the more mercury that can be expressed from the mass in the filling process, the less change is there in the mass after the filling is completed. For my methods in obtaining the desired result I am entirely indebted to Dr. Howe. We do not find that the formula makes any great difference, so long as there is a reasonable proportion of desirable metals in the alloy; as, for instance, we use the same method when we use dentalloy, which is probably about 72 silver, as when we use the alloy which we have made up for us by Baker & Co., which is 49 silver, 48 tin, 2 gold, and 1 platinum. In the first place, the cavity must be a *cul de sac*, or pocket. If it is a compound cavity it must be made so by the use of the matrix. The practice of Dr. Howe and myself is to use a very thin steel band encircling the tooth, soldered together with soft solder. The first amalgam inserted is comparatively plastic. In order to increase the dryness of the material we do not squeeze the amalgam through cloth or anything else; we gradually add filings until the material is so dry that it almost crumbles. Even then the mercury works to the top, and as it does we remove the softened mass and add more of the drier material until the last pellet added is almost a powdery mass. It is then possible to work a little mercury to the surface, which we absorb by the addition of precipitate of silver. This last substance was suggested for this purpose several years ago by Dr. Howe. Now, when the mass is completely hard (which it will be as soon as the process of insertion is finished), the matrix is in most cases immediately removed and the filling trimmed by cutting away any surplus with sharp instruments. As crystallization is not yet complete, it is better to polish at another sitting. I have seen fillings put in in this way that had perfect margins ten or twelve years after their insertion, and these fillings have been of different formulæ, which would indicate that with any reasonable formula the chief thing is the careful extraction of the mercury in the packing, and the proper manipulation in inserting.

I have with me to-night some glass tubes which have been filled by me according to the above-described process,—some in January, 1902, and some last week. All have been recently left to lie in a bath of carmine ink, and you can see for yourselves how much leakage has followed.

The unmarked tube, which shows a slight evidence of leakage, was filled in 1902, and is from a sample of "Columbian A" alloy, which it is claimed has sixty per cent. silver.

I. Columbian D alloy: Sixty-seven per cent. silver, made in 1902; no leakage.

II. Bement's alloy: Silver, 49.85; tin, 49.55; zinc, 0.45; gold, 0.15; made in 1902; leaks.

IV. Flagg's submarine: Silver, 60; tin, 33; copper, 7 (according to his later formula); made in 1902; leaks in spots.

V. True dentalloy: Probably over seventy per cent. silver, mixed according to directions; alloy, 9; mercury, 11; gradually added filings until proportion was filings, 12; mercury, 11; made in 1902; no leakage.

VI. True dentalloy made according to directions after method proposed by Dr. Black: Slight leakage at margins; very much softer than the two made by the other method; done May 9, 1905.

VII. An alloy made for Dr. Howe which we use regularly: Silver, 49; tin, 48; gold, 2; platinum, 1; packed by the method I have described by absorbing surplus mercury, by working in more filings, but no precipitated silver used; no leakage; made May 9, 1905.

VIII. Same as No. VII. was made, except precipitated silver added to finally absorb the last mercury that worked to the surface; no leakage; made May 9, 1905.

IX. True dentalloy made by the same process as No. VIII.; no leakage, and much harder than No. VI.

There is an interesting and instructive paper on amalgams by Dr. St. George Elliott in the April *Dental Cosmos,* which shows a shrinkage in all amalgams sooner or later, and he says that he has little hope that a good reliable amalgam will be produced in the immediate future because of the impossibility of getting pure metals in our alloys and the difficulty of getting a proper mixture of the metals in the melting process.

If mechanical tests made outside the mouth were conclusive it might be so, but it is quite probable that what Mr. Fletcher said in 1875 about external experiments being of little value in our clinical experience is quite as true now, and it seems to me that more care in the manipulation of any ordinary amalgam of known formula might at least contribute to much superior work than the rough, unfinished, and slovenly looking fillings of this material that we so often encounter now.

It is unfortunate that I have not had time to look up the work of Drs. Bogue and Hitchcock (*Transactions of the New York Odontological Society,* 1874).

REFERENCES.

Mr. Fletcher: New York Odontological Society Transactions, 1875.
Dr. J. Foster Flagg: Plastics and Plastic Fillings.
New York Odontological Society Transactions, 1878.
Dr. G. V. Black: Dental Cosmos, 1895–96.
Dr. J. Morgan Howe: New York Odontological Society Transactions, 1894.
Mr. Charles Tomes: Transactions of the British Odontological Society, 1894–95, 1895–96.
Mr. George Cunningham and Mr. Baldwin: Transactions of the British Odontological Society, 1895–96.
Dr. St. George Elliott: Dental Cosmos, April, 1905.

